# The Meaning and Reliability of Minimal Important Differences (MIDs) for Clinician-Reported Outcome Measures (ClinROMs) in Dermatology—A Scoping Review

**DOI:** 10.3390/jpm12071167

**Published:** 2022-07-18

**Authors:** Reinhart Speeckaert, Arno Belpaire, Sandrine Herbelet, Marijn M. Speeckaert, Nanja van Geel

**Affiliations:** 1 Department of Dermatology, Ghent University Hospital, 9000 Ghent, Belgium; arno.belpaire@uzgent.be (A.B.); sandrine.herbelet@ugent.be (S.H.); nanja.vangeel@uzgent.be (N.v.G.); 2 Department of Nephrology, Ghent University Hospital, 9000 Ghent, Belgium; marijn.speeckaert@ugent.be

**Keywords:** minimally important difference, MID, minimally important change, MIC, psoriasis, atopic dermatitis, vitiligo, dermatomyositis

## Abstract

Background: Clinician-reported outcome measures (ClinROMs) are frequently used in clinical trials and daily practice to evaluate the disease status and evolution of skin disorders. The minimal important difference (MID) represents the smallest difference that decreases the disease impact enough to make a treatment change worthwhile for patients. As no clear guidance exists on the preferred method to calculate MIDs for ClinROMs, we evaluated how the published values for different skin disorders should be interpreted. Methods: A systematic search was performed for MIDs of ClinROMs that focus on skin disorders and/or symptoms. The results of the questions in the credibility instrument for MIDs of Devji et al., 2020 were analyzed to gain insights into the meaning of these MIDs. Results: 29 MIDs were identified. The most common skin diseases were atopic dermatitis/eczema, followed by bullous disorders and psoriasis. A minimal important difference from the patients’ perspective was determined in 31% of the cases. However, in 41.4% of the cases, it concerned a substantial rather than a minimal difference in disease severity rated by physicians. Over half (55.1%) of the studies contained an inadequate number of patients (n < 150). MID values increased substantially in patients with severe compared to mild disease. Conclusions: MIDs of ClinROMs for skin disorders should be carefully interpreted due to the substantial differences in methodology between the studies. There is an urgent need for a consensus method to report reliable MIDs. Otherwise, this lack of uniformity could not only affect the design and conclusion of clinical trials but also skew treatment decisions.

## 1. Introduction

Clinician-reported outcome measures (ClinROMs) are important instruments to assess disease status and measure the outcome of interventions. Thresholds in ClinROMs such as the eczema area and severity index (EASI) are used as reimbursement criteria for biologics. Most ClinROMs are designed to detect small differences in disease severity. However, very small differences may not be large enough to change the quality of life or the experienced disease burden of patients (e.g., affected body surface area of 15% that decreases to 14%) [1]. This is why the concept of the minimal (clinically) important difference [M(C)ID] was created, defined as the smallest difference in an outcome measure that is perceived to be important by patients [2]. Values exceeding the MID are often used in clinical trials as proof that a new intervention is worthwhile for patients. This can influence the reimbursement decision about new treatments. Sample size calculations of these trials are also based on this MID value. Additionally, MIDs can be used in clinical practice to guide therapeutic decisions [3,4].

Although there has been debate about what constitutes an ‘important’ difference, the most widely accepted definition of the MID remains the statement of Jaeschke et al. 1989, which defines the MID as “*the smallest difference that would mandate, in the absence of troublesome side effects and excessive cost, a change in the patient’s management”* [5]. To determine the MID value, patients are asked a question (=anchor) about how they feel about the change in their health state, with answer options that reflect small incremental changes such as “much worse”, “somewhat worse”, “the same”, “somewhat better”, and “much better”. The MID is then calculated as the average difference between “the same” and “somewhat worse” or “somewhat better”. Another statistical method is to use a receiver operating characteristic (ROC) analysis. It is recommended to use multiple anchors and, in addition to the opinion of the patient, the physicians’ perspective can also be included. Sometimes the MID is approached by a mathematical calculation based on the variation in the scores of the outcome measure (standard deviation, standard error of measurement, effect size). This is called the distribution-based approach of the MID, which offers additional information, although it deviates from the assumption that the MID is determined by the experience of the patient [3,4,5].

Some authors prefer different terms than the MID, such as “clinically important change” or “meaningful change”. Given the differences in terminology and in methods to determine the MID, the interpretation of MID values can become confusing and even misleading. This may result in wrong conclusions about the efficacy of treatments. Our goal was to evaluate how the published MID values for ClinROMs in dermatology should be interpreted. We also checked if any credibility criteria (such as sample size) were met, which would validate the use of these MID values.

## 2. Materials and Methods

**Figure 1:**
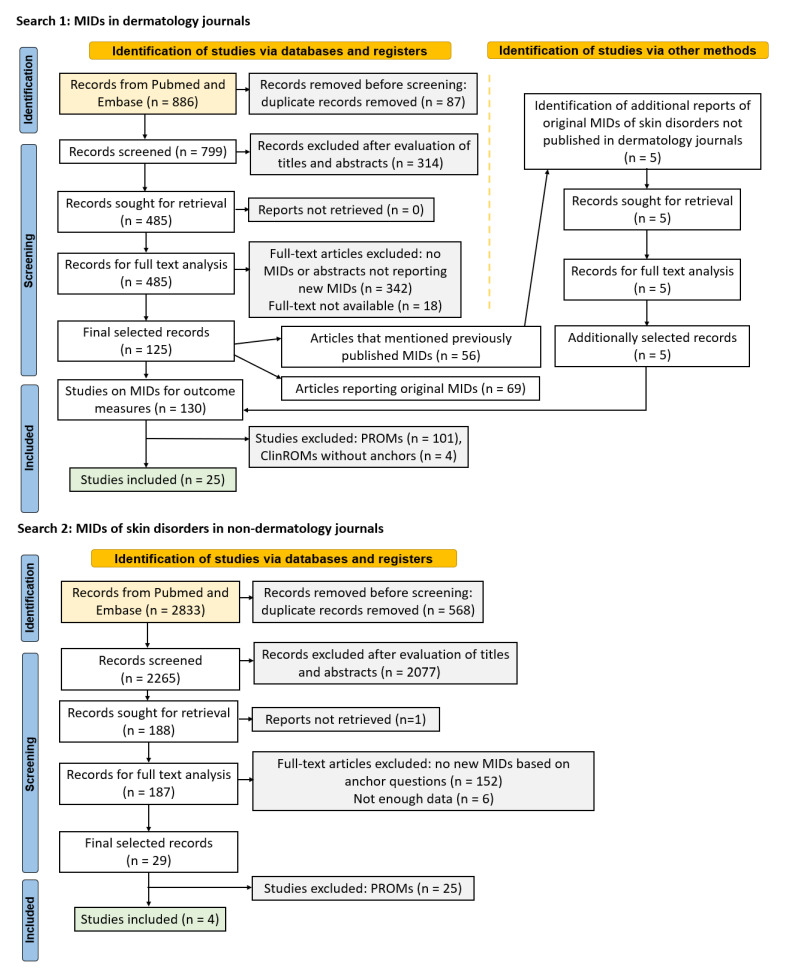
PRISMA flow chart.

A systematic search was done to capture articles on MID values of ClinROMs in the field of dermatology. The PRISMA flow charts are presented in Figure 1, and the search queries are added in the Supplementary Material. The first search was conducted in dermatology journals in Pubmed and Embase from inception to 29 December 2021. A supplementary search was performed in non-dermatology journals based on a list of the most common skin disorders and symptoms (Supplementary Material). Only original research articles determining MIDs by the anchor-based approach were selected. Reviews and studies with arbitrarily set MIDs were excluded. Only the patient-responded anchor was evaluated when anchors were answered by both patients and physicians, because patient evaluation is the preferred method for assessing the MID [4]. Only poster abstracts calculating new MIDs were selected.

An instrument to rate the credibility of MIDs for patient-reported outcome measures (PROMs) was developed by Devji et al., 2020 [6]. As its use has not been validated for ClinROMs, we did not perform a credibility analysis but used the five questions in the instrument of Devji et al. to get more insights into the meaning and reliability of MIDs for ClinROMs. The answers were evaluated according to the provided instructions [6]. The last question of the instrument “*Does the threshold or difference between groups on the anchor used to estimate the MID reflect a small but important difference?*” was split into two questions. This was done to learn more about whether (1) small differences were measured and whether (2) the significance of this difference was considered. This distinction was based on the key aspects of MIDs identified by Terwee et al., 2021 [7]. Instructions for scoring both questions are added in the Supplementary Material. Based on the answers, a statement was made how each MID should be interpreted. In addition, we discussed whether the MID was reliable based on the number of patients and the anchor choice [=good correlation between the (change in the) anchor and the change in the ClinROM].

## 3. Results

Twenty-nine MIDs for ClinROMs were identified. Most MIDs were found for atopic dermatitis and eczema (n = 8), followed by bullous disorders (n = 5), psoriasis (n = 3), vitiligo (n = 2), dermatomyositis (n = 2), localized scleroderma (n = 2), hidradenitis suppurativa (n = 1), lupus (n = 1), sarcoidosis (n = 1), and cellulite (n = 1). Thirteen out of twenty-nine (44.9%) MIDs came from studies fully or partially funded by pharmaceutical companies.

### 3.1. The Characteristics of MIDs for ClinROMs

#### 3.1.1. Methodology: How Is the Minimal Important Difference Determined?

In 13/29 (44.8%), the opinion of the patient defined the minimal difference, whereas in 16/29 (55.2%), the perspective of the physician was used (Table 1). In 3/29 (10.3%), the disease impact or quality of life was mentioned in the anchor question. In 9/29 (31.0%) and 16/29 (55.2%), a global severity scale was used, which was answered by patients or physicians, respectively. In 15/29 (51.7%), answer options for the anchor question were available that reflected small differences such a “mild disease” or “little improvement”.

#### 3.1.2. Reliability: Is the Sample Size Adequate, and Is the Anchor Selection Appropriate?

A substantial number of studies (16/29; 55.1%) had an inadequate number of patients (n < 150). This makes the MID value less reliable. The patient group should encompass enough patients with different grades of disease severity to be representative. Correlations between the (change in the) anchor and the change in the PROM are good proof that the anchor is appropriate. In 7/29 (24.1%), good correlations were reported and in 5/29 (17.2%), there were moderate correlations. The majority of studies failed to report these correlations (17/29; 58.6%).

### 3.2. Results for Different Skin Disorders

#### 3.2.1. Atopic Dermatitis/Eczema

The eczema area and severity index (EASI) has two established MIDs ranging from 6.6 to 10.9 (Table 2) [9,10]. The lowest value was obtained when the investigator global assessment (IGA) was used as an anchor, whereas the highest value was obtained using the patient-reported global assessment (PGA) of atopic dermatitis (AD) severity. Although no correlations were performed between the EASI and the anchor, both studies used a global severity scale as an anchor, which is likely to be a good measure for a PROM (EASI) representing the severity of the disease.

Two MIDs were also published on the hand eczema severity index (HECSI), albeit with markedly different values (MID: 4.5–7.1 versus 10.5–30.2) [11,12]. However, both studies found that the MID of the HECSI varies significantly depending on the severity of the hand eczema at baseline. The different disease severity of the patient groups in both studies (HECSI: 29.3 vs. 45.1) likely contributed to the broad range in MID values [11,12]. In addition, for the SCORing Atopic Dermatitis (SCORAD), two MIDs were calculated. Schram et al., 2012 reported a value of 8.7, with the physician rating a global severity scale, compared to 16.6 by Silverberg et al., 2021, with the patient responding to the anchor [9,10]. The SCORAD changes according to the AD severity from 2.7–15.8 in mild AD to 17.5–23.3 in moderate AD and 22.3–29.2 in severe AD [10].

#### 3.2.2. Bullous Skin Disorders

MIDs were found for the autoimmune bullous skin disorder intensity score (ABSIS), bullous pemphigoid disease area index (BPDAI), pemphigus disease area index (PDAI), and physician-reported outcome measure epidermolysis bullosa disease activity and scarring index (EBDASI). In all studies, the physicians’ opinion was used, and the number of patients was very low. This is possibly due to the relatively low incidence of bullous pemphigoid, pemphigus, and epidermolysis bullosa [13,14,15]. Multiple observations of the same patients, however, were performed to increase the number of ratings.

#### 3.2.3. Psoriasis

Two studies found a very similar MID for the 3-item PGA in psoriasis (MID = 0.52 vs. 0.55) [16,17]. Both studies used the patients’ perspective and scored very favorable according to the credibility criteria of Devji et al., 2020 developed for PROMs [6]. This MID can therefore reliably be used in future studies.

#### 3.2.4. Vitiligo

A clinically meaningful change for the vitiligo area scoring index (VASI) was determined, although the authors did not mention the word “minimal”, and it is likely that the authors had no intention of calculating an MID, as the meaningful change was set to only include patients with much or very much improvement. For the vitiligo extent score (VES), the minimal change in vitiligo extent that patients can perceive was investigated and not the importance of this change [18]. The minimal change that is important from a patients’ or physicians’ perspective has not been studied yet for the VES and the VASI.

#### 3.2.5. Dermatomyositis

MIDs are available for the cutaneous dermatomyositis disease area and severity index (CDASI) and the cutaneous dermatomyositis disease area and severity index activity (CDASI-A) [19,20]. However, the manuscript does not explain why a 2-point change in the PGA was used to assess the MID for the CDASI rather than a 1-point change [19].

#### 3.2.6. Localized Scleroderma

One study is available for the modified localized scleroderma skin severity index (mLOSSI) and physician global assessment of disease activity (PGA-A) [21]. The limited number of patients (n = 29) requires more research for validating the published values.

#### 3.2.7. Other Skin Disorders 

For hidradenitis suppurativa, data are published on the hidradenitis suppurativa clinical response (HiSCR) [22]. For sarcoidosis (cutaneous sarcoidosis activity and morphology instrument (CSAMI)), lupus (cutaneous lupus disease area and severity index activity score (CLASI-A)), and cellulite (photonumeric cellulite severity scale (CR-PCSS)), the number of patients was too small to be considered as reliable for determining MIDs [23,24,25].

**Table 2:** Scoring of the credibility of MIDs for physician-reported outcome measures.

	Is the Patient or Necessary Proxy Responding Directly to the Anchor and the Physician to the Outcome Measure?	Is the Anchor Easily Understandable and Relevant for Patients or Necessary Proxy?	Has the Anchor Shown Good Correlation with the Patient-Reported Outcome Measure?	Is the MID Precise? (95% Confidence Interval or Number of Patients Included in the Estimation)	Does Anchor Used to Estimate the MID Reflect a Small Difference/Change?	Does the Anchor Question Assess the Importance According to the Patient and Not Only a Change in Clinical Signs/Symptoms?	- What Does This MID Mean? - Is the Value Reliable? ^1^
**Atopic Dermatitis/Eczema**							
**Eczema Area and Severity Index (EASI)**							
Schram et al., 2012 [9]	No, physician			N = 42 (239 observations)		No, IGA	- Minimal difference in physicians’ assessment of disease severity (MID = 6.6) - No, correlation is missing and moderate number of patients.
Silverberg et al., 2021 [10]							- Minimal difference in patients’ rated disease severity (MID_improvement_ = 10.9) - Unclear, correlation is missing.
**Hand Eczema Severity Index (HECSI)**							
Yüksel et al., 2021 [11]							- Minimal difference in patients’ rated disease severity (MID = 4.5–7.1; mild: 1.5; moderate-severe: 8.9–9.5); - Unclear, correlation is missing.
Oosterhaven et al., 2020 [12]	No, physician					No, score by physicians	- Minimal difference in physicians’ assessment of disease severity (MID = 10.5–30.2; low baseline HECSI: 5.5–10.7; High baseline HECSI: 19.0–46.9) - Yes, but only from a physicians’ perspective.
**Investigator’s Global Assessment (IGA) x Body Surface Area in Children and Adults with Atopic Dermatitis**							
Silverberg et al., 2021 [26]	No, physician		^2^			No, global severity (physician)	- Minimal difference in physicians’ assessment of disease severity (MID = 1.0) - Yes, but only from a physicians’ perspective.
**Investigator Global Assessment for Atopic Dermatitis (vIGA-AD™)**							
Simpson et al., 2022 [27]							- Minimal difference in patients’ rated disease severity (MID = −1.00) - Unclear, correlation is missing.
**Occupational Contact Dermatitis Disease Severity Index (ODDI)**							
Ofenloch et al., 2015 [28]	No, physician		^2^ 0.48			No, PGA	- Minimal difference in physicians’ assessment of disease severity (MID = 1.29) - Yes, but only from a physicians’ perspective.
**Rajka–Langeland Severity Score**							
Silverberg et al., 2021 [29]							- Minimal difference in physicians’ assessment of disease severity (−0.9–−1.2) - Unclear, correlation is missing.
**SCORing Atopic Dermatitis (SCORAD)**							
Schram et al., 2012 [9]	No, physician			N = 42 (239 observations)		No, IGA	- Minimal difference in patients’ rated disease severity (MID = 8.7) - No, correlation is missing and moderate number of patients
Silverberg et al., 2021 [10]							- Minimal difference in patients’ rated disease severity (MID = 16.6; mild AD: 2.7–15.8; moderate AD: 17.5–23.3; severe AD: 22.3–29.2); - Unclear, correlation is missing.
**Objective SCORing Atopic Dermatitis (O-SCORAD)**							
Silverberg et al., 2021 [10]							- Minimal difference in patients’ rated disease severity (MID = 13.0; mild AD: 1.5–11.7; moderate AD: 17.5–23.3; severe AD: 22.3–29.2); - Unclear, correlation is missing.
**Bullous Skin Disorders**							
**Autoimmune Bullous Skin Disorder Intensity Score (ABSIS)**							
Hanna et al., 2017 [13]	No, physician		0.48	N = 28	PGA ≥ 2 or Likert ≥ 3 considered as minimally changed	No, score by physicians	- A substantial difference in physicians’ assessment of disease severity (MID= +/−8.5) - No, small number of patients.
Wijayanti et al., 2017 [14]	No, physician			N = 27 (108 observations)	Broad categories: improved, stable, or deteriorated	No, score by physicians	- A substantial difference in physicians’ assessment of disease severity [MID: 8.6 (improvement); 4 (deterioriation)] - No, small number of patients.
**Bullous Pemphigoid Disease Area Index (BPDAI)**							
Wijayanti et al., 2017 [14]	No, physician			N = 27 (108 observations)	Broad categories: improved, stable, or deteriorated	No, score by physicians	- A substantial difference in physicians’ assessment of disease severity [MID = 4 (improvement); 3 (deterioration)] - No, small number of patients.
**Pemphigus Disease Area Index (PDAI)**							
Hanna et al., 2017 [13]	No, physician		−0.46	N = 28	PGA ≥ 2 or Likert scale ≥ 3 considered as minimally changed	No, score by physicians	- A substantial difference in physicians’ assessment of disease severity (MID = +/−3) - No, small number of patients
**Physician-Reported Outcome Measure: Epidermolysis Bullosa Disease Activity and Scarring Index (EBDASI)**							
Jain et al., 2017 [15]	No, physician			N = 29	Broad categories: 3-point Likert	No, score by physicians	- A substantial difference in physicians’ assessment of disease severity [MID: 3 (deterioration); 9 (improvement)] - No, small number of patients and no correlation
**Cellulite**							
**Photonumeric Cellulite Severity Scale (CR-PCSS)**							
Cohen et al., 2020 [25]	No, physician		−0.65	N = 76	Smallest categories: improved or worse.	No, score by physicians	- A substantial difference in physicians’ assessment of disease severity (MID = 1.0) - No, small number of patients
**Dermatomyositis**							
**Cutaneous Dermatomyositis Disease Area and Severity Index Activity (CDASI-A)**							
Ahmed et al., 2020 [20]				N = 103			- Minimal difference in patients’ experienced disease impact/disease-related quality of life (MID: 7.86 (symptoms); MID: 10.29 (emotions) - No, correlation is missing and moderate number of patients
**Cutaneous Dermatomyositis Disease Area and Severity Index (CDASI)**							
Anyanwu et al., 2015 [19]	No, physician			N = 128	2-point change in PGA-VAS considered as minimally changed	No, PGA	- A substantial difference in physicians’ assessment of disease severity (MID = 4–5) - No, moderate number of patients and correlation is missing
**Hidradenitis Suppurativa**							
**Hidradenitis Suppurativa Clinical Response (HiSCR)**							
Kimball et al., 2014 [22]			^2^ −0.27–−0.47	N = 138	Broad categories in the ClinROM (0- < 30; 30- < 40; 40- < 50…)		- Minimal difference in patients’ experienced disease impact/disease-related quality of life. However due to the broad categories in the ClinROM, the change may be considered as substantial. (MID= 0.77–2.72); - No, moderate number of patients
**Localized Scleroderma**							
**Modified Localized Scleroderma Skin Severity Index (mLOSSI)**							
Kelsey et al., 2013 [21]	No, physician			N = 29	Broad categories: Active or inactive	No, activity classification (physcian)	- A substantial difference in physicians’ assessment of disease severity (MID = 6 (4–8)) - No, low number of patients
**Physician Global Assessment of Disease Activity (PGA-A)**							
Kelsey et al., 2013 [21]	No, physician			N = 29	Broad categories: Active or inactive	No, activity classification (physcian)	- A substantial difference in physicians’ assessment of disease severity (MID = 41 (34–51)) - No, low number of patients
Lupus							
**Cutaneous Lupus Disease Area and Severity Index Activity Score (CLASI-A)**							
Chakka et al., 2021 [24]				N = 8	Threshold in a PROM (Skindex-29 E: 9.38; Skindex-29 S: 7.37)		- A substantial difference in patients’ experienced disease impact/disease-related quality of life (MID = 3.3–7.4) - No, very low number of patients
**Psoriasis**							
**Three-Item Physician-Global Assessment: PGA (Psoriasis) (Erythema, Induration and Scaling, Individually, on a Five-Point Scale (from 0 = no symptom to 4 = severe)**							
Cappelleri et al., 2013 [16]			^2^	N = 197			- Minimal difference in patients’ rated disease severity (MID = 0.52; 95% CI: 0.47–0.56) - Yes
Duffin et al., 2019 [17]			^2^				- Minimal difference in patients’ rated disease severity (MID = 0.55; 0.45–0.56) - Yes
**Simplified Psoriasis Index (SPI)**							
Chularojanamontri et al., 2014 [30]	No, physician			N = 100	PASI-50 but not PASI-75	No, PASI	- A substantial difference in physicians’ assessment of disease severity (MID= 5.25–7.57) - No, moderate number of patients
**Sarcoidosis**							
**Cutaneous Sarcoidosis Activity and Morphology Instrument (CSAMI)**							
Noe et al., 2020 [23]	No, physician			N = 41	Broad categories: improved-no change-worsened	No, physician change rating (PCR)	- A substantial difference in physicians’ assessment of disease severity (MID = 5) - No, low number of patients
**Vitiligo**							
**VASI (Vitiligo Area Scoring Index)**							
Hamzavi et al., 2021 [31]			0.45	N = 157	Very much and much improved = meaningfully changed		- A substantial difference in physicians’ assessment of disease severity (MID = 57% improvement of the facial-VASI; 42% improvement of the total-VASI) - Yes, but for a substantial and not a minimal difference
**Vitiligo Extent Score (VES)**							
Uitentuis et al., 2021 [18]						No, only improvement in vitiligo extent	- Minimal difference in patients’ rated symptoms (MID = 0.5%) - Yes, for a subjectively significant difference (= smallest difference that a patient can detect), but not for an MID (only symptoms are assessed and no correlation) [4].

Dark green: “definitely yes”; light green: “to a great extent”; yellow: “not so much”; red: “definitely no”; black: “impossible to tell”. ^1^ This conclusion is not an evaluation of the study quality per se but only a rating of the reported MID. ^2^ The reported correlation is not between the change of the PROM and the change of the anchor (or the transition rating anchor) or it is unclear whether it concerns baseline, follow-up, or changed values.

## 4. Discussion

The treatment options for several inflammatory and malignant skin disorders are increasing rapidly due to the rise of targeted treatments. In clinical trials, scoring instruments completed by physicians (=ClinROMs) are often used to assess and follow the disease severity. These ClinROMs (e.g., EASI, SCORAD…) can be included in the criteria of drug reimbursement and implemented in clinical practice to evaluate disease evolution over time. As very small differences (e.g., a very small decrease in the affected skin area) may not change the perceived disease burden in a meaningful way, the MID concept has been created to represent the smallest difference in disease status that justifies a change in treatment. Methodologists have outlined the general rules for determining MIDs for PROMs, and their credibility can be rated based on several criteria [6]. However, it is unclear whether the same rules also apply to ClinROMs. This lack of consensus has likely contributed to the large variety in methodological approaches found in this review. Future efforts should focus on the minimum criteria necessary to publish a credible MID for ClinROMs, as guidance is currently missing.

In this study, we found that the published MIDs for ClinROMs have different meanings depending on the used methodology. In 12/29 (41.4%) cases, the MIDs for ClinROMs represented a substantial difference in disease severity assessed by physicians. This violates two basic assumptions from the definition of the MID by Jaeschke et al., namely, that the difference should be minimal and is based on the experience of the patient [5]. In 5/29 ClinROMs (17.2%), it concerned a minimal difference in disease severity rated by physicians. In all other cases, the patient responded to the anchor questions. Patients assessed a minimal difference in disease severity in 7/29 (24.1%), in 2/29 (6.9%) a minimal difference in impact/quality of life, in 1/29 (3.4%) a minimal difference in symptoms, and in 1/29 (3.4%) a substantial difference in disease severity. We added the interpretation of each MID to Table 2. From the eight studies that did not include the word “minimal” but terms such as “meaningful change” or “clinically important difference”, four studies (50%) did assess a minimal difference. Finally, for 58.6%, no data are available on the correlation between the (change in the) anchor and the (change in the) ClinROM. As such, it is impossible to check whether the anchor reflects what is measured by the ClinROM and is appropriate to determine the MID.

Patient groups with variable disease severity had markedly different MID values (e.g., SCORAD, HECSI) [10,12]. Future research should clarify not only the diagnosis but also the disease severity to which the reported MID applies. If an MID is approached from the physicians’ perspective, the MID value can be influenced by the physician’s speciality and experience with the disease [32]. A study on abdominal aortic aneurysms found that variability in the characteristics of physicians could introduce variance in the sample size of 116 to 3015 when calculated based on the MID [32]. In most studies of MIDs, the clinical experience of the physicians is not mentioned, which could offer more insights to the interpretation of the MIDs.

Most MIDs have been published on AD/eczema. However, in the case of the EASI, SCORAD, and HECSI, which each had two published MID studies, the MIDs were substantially different and increased in patients with severe AD [9,10,11,12]. For psoriasis, the 3-item PGA has two well-conducted publications with very similar MIDs [16,17]. The ABSIS for bullous pemphigoid also has two comparable MIDs, although the small number of patients requires further investigation [13,14]. There is still work to be done to determine the MIDs of the vitiligo extent instruments (VES and VASI) [18,31]. Localized scleroderma, lupus, sarcoidosis, and cellulite all have a clinically important difference for a ClinROM that represents a substantial rather than a minimal difference, whereas an actual MID is present for hidradenitis suppurativa. 

In conclusion, there is an urgent need for a consensus on what constitutes an MID for a ClinROM. The current publications on skin disorders display a variety in interpretations, leading to a confusing collection of MIDs with different meanings. This can lead to misleading calculations of sample sizes for clinical trials and values that may fail to approximate the change needed to justify a treatment change from the patients’ perspective. 

## Figures and Tables

**Table 1:** Analysis of the characteristics of MIDs for ClinROMs.

Credibility Instrument of Devji et al., 2021 [6]	
**1. Is the patient or necessary proxy responding directly to the anchor and the physician to the outcome measure?**	
- Patient or proxy responding	13/29 (44.8%)
- Physician/investigator responding	16/29 (55.2%)
- Different patient group responding	0/29 (0%)
**2. Is the anchor easily understandable and relevant for patients or necessary proxies?**	
- Avoidance of difficult medical terminology, statements deemed adequate for their purpose	13/13 (100%)
- Global assessments of change or global ratings of disease severity or disease activity that are generally accepted as easy to understand for patients	7/13 (76.9%)
- Validated PROMS with confirmed comprehensibility for patients	3/13 (23.1%)
**3. Has the anchor shown a good correlation with the PROM?**	
- Good correlation with the PROM (r ≥ 0.5)	7/29 (24.1%)
- Moderate correlation with the PROM (r ≥ 0.3–0.5)	5/29 (17.2%)
- Low correlation with the PROM (r < 0.3)	0/29 (0%)
- No correlation mentioned	17/29 (58.6%)
**4. Is the MID precise? (number of patients included)**	
- Very high number of patients	9/29 (31.0%)
- High number of patients	4/29 (13.8%)
- Less than adequate number of patients	6/29 (20.7%)
- Insufficient number of patients	10/29 (34.5%)
**5. Does the threshold or difference between groups on the anchor used to estimate the MID reflect a small but important difference?**	
**5a. Does the anchor used to estimate the MID reflect a small difference?**	
- Small differences taken into account (most used terms: “mild”, “little”)	15/29 (51.7%)
- No small differences taken into account	14/29 (48.3%)
**5b. Does the anchor question assess the importance according to the patient and not only a change in clinical signs/symptoms?** (Anchor questions were evaluated using the original definition of the MID, which indicates that the patient’s perception should be included [8])	
- Anchor questions handling the impact of the disease on the quality of life, tolerability of the disease, functional implications, and emotions (=> highly likely to accurately reflect important changes for the patients)	3/29 (10.3%)
- Anchor questions about the severity of the disease/symptoms from a patients’ perspective {Rating the severity of the disease was considered a questionable (but acceptable) approach for stratifying disease states that mandate a change in treatment)	9/29 (31.0%)
- Anchor questions assessing a small detectable change in symptoms rather than a clinically important change from a patients’ perspective	1/29 (3.4%)
- Anchor questions not answered by patients, but assessing the global severity of the disease	16/29 (55.2%)

## Supplementary Materials

The following supporting information can be downloaded at: https://www.mdpi.com/article/10.3390/jpm12071167/s1, Supplementary material: Search queries for the scoping review.
