# Supplementary material for: The Meaning and Reliability of Minimal Important Differences (MIDs) for Clinician-Reported Outcome Measures (ClinROMs) in Dermatology—A Scoping Review
Source: J Pers Med. 2022 Jul 18;12(7):1167. doi: 10.3390/jpm12071167 (PMC9321211; doi:10.3390/jpm12071167)
Supplement: Supplementary file 1 [file jpm-12-01167-s001.zip › jpm-1810507-supplementary.pdf]

Supplementary material: Search strategy for the scoping review.

Pubmed and Embase:

Search query 1: Identification of MIDs of PROMs in dermatology journals

#1. All fields:

'minimal clinically important difference' OR ‘minimal important difference’ OR ‘minimal important change’ OR 'minimal clinically important change' OR ‘MCID’ OR ‘MCIC’ OR ‘minimal important difference’ OR ‘Clinically important difference’ OR ‘Clinically meaningful’ OR ’Clinically relevant change’ OR ‘Clinically significant improvement’ OR ‘Minimal clinical important difference’ OR ‘Minimal clinically important change’ OR ‘Minimal clinically important improvement’ OR ‘Minimal clinically important score-difference’ OR ‘Minimal clinically significant difference’ OR ‘Minimal clinically significant difference’ OR ‘Minimal important change’ OR ‘Minimal important deterioration’ OR ‘Minimal important difference’ OR ‘Minimal important improvement’ OR ‘Minimal patient perceivable’ OR ‘Minimally clinical important difference’ OR ‘Minimally clinically identifiable difference’ OR ‘Minimally clinically important change’ OR ‘Minimally clinically important difference’ OR ’ Minimally important change’ OR ‘Minimally important difference’ OR ‘Minimally important percent change’ OR ‘Minimum clinical important difference’ OR ‘Minimum clinically important change’ OR ‘Minimum clinically important difference’ OR ‘Minimum clinically important improvement’ OR ‘Minimum clinically significant change’ OR ‘Minimum clinically significant difference’ OR ‘Minimum important change’ OR ‘Minimum important difference’ OR ‘Meaningful improvement’ OR ‘Meaningful change’

AND

#2. Journal:

Journal of the American Academy of Dermatology[Journal] OR JAMA Dermatology OR ‘British Journal of Dermatology[Journal] OR Journal of Investigative Dermatology[Journal] OR American Journal of Clinical Dermatology[Journal] OR Contact Dermatitis[Journal] OR Journal of the European Academy of Dermatology and Venereology[Journal] OR Journal der Deutschen Dermatologischen Gesellschaft[Journal] OR Dermatology[Journal] OR Burns and Trauma[Journal] OR Dermatitis[Journal] OR Advances in Wound Care[Journal] OR Pigment Cell and Melanoma Research[Journal] OR Pigment Cell[Journal] OR Journal of Dermatological Science[Journal] OR Acta Dermato-Venereologica[Journal] OR Mycoses[Journal] OR Lasers in Surgery and Medicine[Journal] OR Journal of Dermatology[Journal] OR Experimental Dermatology[Journal] OR Wound Repair and Regeneration[Journal] OR Melanoma Research[Journal] OR Clinics in Dermatology[Journal] OR Skin Pharmacology and Physiology[Journal] OR Dermatologic Clinics[Journal] OR Clinical and Experimental Dermatology[Journal] OR Dermatologic Surgery[Journal] OR Journal of Dermatological Treatment[Journal] OR European Journal of Dermatology[Journal] OR International Wound Journal[Journal] OR Dermatology and Therapy[Journal] OR Photodermatology, Photoimmunology and Photomedicine[Journal] OR Archives of Dermatological Research[Journal] OR International Journal of Cosmetic Science[Journal] OR Journal of Tissue Viability[Journal] OR Australasian Journal of Dermatology[Journal] OR Dermatologic Therapy[Journal] OR Burns[Journal] OR International Journal of Dermatology[Journal] OR Journal of Cosmetic Dermatology[Journal] OR Indian Journal of Dermatology, Venereology and Leprology[Journal] OR Clinical, Cosmetic and Investigational Dermatology[Journal] OR Skin Research and Technology[Journal] OR Advances in Skin and Wound Care[Journal] OR Journal of Cosmetic and Laser Therapy[Journal] OR Journal of Drugs in Dermatology[Journal] OR Journal of Cutaneous Medicine and Surgery[Journal] OR Journal of Wound Care[Journal] OR The International Journal of Lower Extremity Wounds[Journal] OR Italian Journal of Dermatology and Venereology[Journal] OR Dermatology Practical and

Conceptual[Journal] OR Anais Brasileiros de Dermatolog[Journal] OR Journal of Burn Care an[Journal] OR Advances in Dermatology and Allergology[Journal] OR Pediatric Dermatology[Journal] OR Journal of Cutaneous Pathology[Journal] OR Wounds[Journal] OR American Journal of Dermatopathology[Journal] OR Indian Journal of Dermatology[Journal] OR Annals of Dermatology[Journal] OR Cutis[Journal] OR Dermatologica Sinica[Journal] OR Acta Dermatovenerologica Croatica[Journal] OR Journal of Cosmetic Science[Journal] OR Hautarzt[Journal] OR Annales de Dermatologie et de Vénéréologie OR 'Wound Management and Prevention[Journal] OR Leprosy Review[Journal] OR Hong Kong Journal of Dermatology and Venereology[Journal]

Search query 2 (05/05/'22): Identification of MIDs of PROMs of skin disorders in non-dermatology journals

---

=> #1 AND #3 NOT #2

#3. Keywords (All fields) for identifying dermatological conditions

'Acne' or 'Actinic keratosis' or 'Alopecia' or 'Basal cell carcinoma' or 'Bowen' or 'Bullous' or 'Dermatomyositis' or 'Dermatosclerosis' or 'Dermatitis' or 'DRESS' or 'Eczema' or 'Erythema multiforme' or 'Granuloma annulare' or 'Hidradenitis suppurativa' or 'Hyperhidrosis' or 'Itch' or 'Lichen' or 'Lupus' or 'Lyell' or 'melanoma' or 'Melasma' or 'Morphea' or 'Mycosis' or 'Palmoplantar pustulosis' or 'Parapsoriasis or 'Pemphigus' or 'Pityriasis' or 'Prurigo' or 'Pruritus' or 'Pustulosis' or 'Psoriasis' or 'Pyoderma' or 'Rosacea' or 'Sarcoidosis' or 'Skin cancer' or 'Scleroderma' or 'Squamous cell carcinoma' or 'Toxic epidermal necrolysis' or 'Urticaria' or 'Vasculitis' or 'Vitiligo'
